# Usefulness of compressed sensing coronary magnetic resonance angiography with deep learning reconstruction

**DOI:** 10.1007/s11604-025-01830-5

**Published:** 2025-07-07

**Authors:** Kohei Tabo, Tomoyuki Kido, Megumi Matsuda, Shota Tokui, Genki Mizogami, Yoshihiro Takimoto, Masaki Matsumoto, Mitsuharu Miyoshi, Teruhito Kido

**Affiliations:** 1 Department of Radiology, Ehime University Graduate School of Medicine, Shitsukawa, Toon, Ehime Japan; 2 Ehime University Hospital, Shitsukawa, Toon, Ehime Japan; 3 GE HealthCare, Hino, Tokyo Japan

**Keywords:** Ccardiovascular magnetic resonance, Coronary magnetic resonance angiography, Compressed sensing, Deep learning reconstruction

## Abstract

**Purpose:**

Coronary magnetic resonance angiography (CMRA) scans are generally time-consuming. CMRA with compressed sensing (CS) and artificial intelligence (AI) (CSAI CMRA) is expected to shorten the imaging time while maintaining image quality. This study aimed to evaluate the usefulness of CS and AI for non-contrast CMRA.

**Materials and methods:**

Twenty volunteers underwent both CS and conventional CMRA. Conventional CMRA employed parallel imaging (PI) with an acceleration factor of 2. CS CMRA employed a combination of PI and CS with an acceleration factor of 3. Deep learning reconstruction was performed offline on the CS CMRA data after scanning, which was defined as CSAI CMRA. We compared the imaging time, image quality, signal-to-noise ratio (SNR), contrast-to-noise ratio (CNR), and vessel sharpness for each CMRA scan.

**Results:**

The CS CMRA scan time was significantly shorter than that of conventional CMRA (460 s [343,753 s] vs. 727 s [567,939 s], *p* < 0.001). The image quality scores of the left anterior descending artery (LAD) and left circumflex artery (LCX) were significantly higher in conventional CMRA (LAD: 3.3 ± 0.7, LCX: 3.3 ± 0.7) and CSAI CMRA (LAD: 3.7 ± 0.6, LCX: 3.5 ± 0.7) than the CS CMRA (LAD: 2.9 ± 0.6, LCX: 2.9 ± 0.6) (*p* < 0.05). The right coronary artery scores did not vary among the three groups (*p* = 0.087). The SNR and CNR were significantly higher in CSAI CMRA (SNR: 12.3 [9.7, 13.7], CNR: 12.3 [10.5, 14.5]) and CS CMRA (SNR: 10.5 [8.2, 12.6], CNR: 9.5 [7.9, 12.6]) than conventional CMRA (SNR: 9.0 [7.8, 11.1], CNR: 7.7 [6.0, 10.1]) (*p* < 0.01). The vessel sharpness was significantly higher in CSAI CMRA (LAD: 0.87 [0.78, 0.91]) (*p* < 0.05), with no significant difference between the CS CMRA (LAD: 0.77 [0.71, 0.83]) and conventional CMRA (LAD: 0.77 [0.71, 0.86]).

**Conclusion:**

CSAI CMRA can shorten the imaging time while maintaining good image quality.

## Introduction

Cardiovascular magnetic resonance imaging (MRI) is considered an effective method for the assessment of cardiac morphology, cardiac function, and myocardial tissue characteristics [1, 2]. Whole-heart coronary magnetic resonance angiography (CMRA) is a noninvasive method that evaluates coronary artery direction, cardiac abnormalities, and coronary artery disease (CAD) [3]. CMRA has the advantage of the absence of radiation exposure, no need for contrast administration, and being unaffected by calcification. Because CMRA is a free-breathing imaging technique with a synchronized diaphragm and electrocardiogram (ECG), it is relatively easy to perform, even in individuals who have difficulty holding their breath or those with hearing loss. Recently, high-resolution CMRA has demonstrated excellent diagnostic accuracy in the detection of CAD, characterized by high sensitivity and negative predictive value [4]. However, CMRA is generally time-consuming, which limits its clinical use. Furthermore, longer imaging times are more susceptible to patient movement and changes in the heart rate (HR) and respiration, which reduce imaging efficiency and degrade image quality.

Integrating compressed sensing (CS) into CMR allows image reconstruction from a partially filled k-space [5]. Moreover, Nakamura et al. reported that non-contrast CS CMRA could significantly shorten the imaging times when compared with conventional CMRA in healthy volunteers. Nonetheless, while non-contrast CS CMRA provides acceptable visualization, the image quality is slightly inferior to that of non-contrast conventional CMRA [6].

With the widespread use of 3 T MRI, cardiovascular MRI is increasingly performed at 3 T. 3 T MRI has a higher magnetic field strength than 1.5 T MRI and produces images with a higher signal-to-noise ratio (SNR) [7]. Delayed enhancement MRI also improves contrast-to-noise ratio (CNR) in normal and abnormal myocardium [8]. 1.5 T CMRA commonly uses balanced steady state free precession (bSSFP) sequence with high SNR and CNR [9]. However, the use of bSSFP sequence is limited by the fact that 3 T is more susceptible to magnetic field inhomogeneity than 1.5 T and the increased power deposition in the human body. Therefore, 3 T CMRA uses a spoiled gradient echo (GRE) sequence that is resistant to magnetic field inhomogeneity, but the signal in the coronary arteries is lower than SSFP in non-contrast.

On the other hand, artificial intelligence (AI) is being increasingly adopted across various sectors, including healthcare. Deep learning reconstruction (DLR), which is a deep learning-based image reconstruction method using convolutional neural networks, is an AI technique gaining attention owing to its potential in improving image quality by noise reduction in the MRI [10–12]. In cardiovascular MRI, DLR has been reported to improve image quality in high-speed cine MRI by under sampling data [13]. Combining CS and DLR techniques in MRI is expected to shorten the imaging time while maintaining the image quality. However, usefulness of CMRA with CS and AI techniques (CSAI CMRA) has not yet been fully investigated. As a preliminary study, we aimed to evaluate the usefulness of non-contrast CSAI CMRA compared to conventional CMRA and CS CMRA with a 3 T scanner. The primary endpoints were imaging time and image quality.

## Materials and methods

### Study population

Twenty healthy volunteers (20 males) were enrolled. All volunteers provided written informed consent and underwent CS CMRA and conventional CMRA without contrast medium. The age, height, weight, and body mass index of the volunteers ranged from 23 to 43 (mean ± standard deviation [SD], 30.9 ± 5.2) years, 160 to 182 (mean ± SD, 170.8 ± 5.9) cm, 52 to 89 (mean ± SD, 67.2 ± 11.3) kg, and 18.1 to 28.3 (mean ± SD, 22.9 ± 3.0) kg/m^2^. None of the volunteers had any history or risk of heart disease. The study protocol was approved by the hospital’s Institutional Review Board.

### CMRA protocol

All MRI examinations were conducted using a clinical 3 T MR scanner (Signa Architect 3 T; GE Healthcare, Waukesha, WI, USA). CMRA was performed using electrocardiogram-triggered, and navigator-gated techniques. First, Scout images of the heart in the axial, sagittal, and coronal views were acquired. Subsequently, a long-axis cine sequence with ECG triggering was acquired to determine the CMRA data acquisition time. The voltage-specific acquisition time was set by an investigator according to the phase of minimal right coronary artery (RCA) motion by observing the coronary artery stagnation time. The CMRA scans were acquired using a T2-prepared segmented three-dimensional spoiled GRE. Chemical shift selective was used as fat saturation to improve coronary artery delineation and suppress fat-related aliasing artifacts. Conventional CMRA uses parallel imaging (PI) with an acceleration factor of 2. The CS CMRA employed a combination of PI and CS with an acceleration factor of 3. Currently, PI is widely used in clinical MR, but because of the increased noise at higher acceleration factors, it is standardly limited to a factor of approximately 2 accelerations to maintain reliable visualization [6, 14, 15]. Research indicates that CS acceleration factors typically range from 3 to 9 [16]. Since this is a preliminary study and a high acceleration factor is expected to degrade image quality, an acceleration factor of 3 was first considered. The detailed imaging parameters are listed in Table 1. The imaging order of CS and conventional CMRA scans was randomized. Nitroglycerin sublingual spray (0.3 mg) was administered before the CMRA scan. Respiratory motion was detected by placing the navigator on the right hemidiaphragmatic dome. An edge-detection algorithm was subsequently used to identify the location of the lung-liver inface. The ratio detected by the navigator within the window setting was used as the acceptance rate.

**Table 1:** Image parameters

	Conventional CMRA	CS CMRA
Sequence type	Spoiled gradient echo	Spoiled gradient echo
Fat saturation	Chemical shift selective	Chemical shift selective
TR/TE (ms)	4.7/2.1 (minimum full)	4.7/2.1 (minimum full)
FOV (mm)	400 × 240	400 × 240
Actual voxel size (mm)	1.3 × 1.3 × 1.5	1.3 × 1.3 × 1.5
Reconstruction voxel size (mm)	0.8 × 0.8 × 0.5	0.8 × 0.8 × 0.5
Temporal resolution (ms)	adapted to the individual heart rate of the subject 100 (70, 100)	adapted to the individual heart rate of the subject 100 (70, 100)
Bandwidth (Hz/pixel)	62.5	62.5
Acceleration factor	2.0	3.0
Acceptance window (mm)	± 3.0	± 3.0

Temporal resolution is presented as the median (first quartile, third quartile)

*CMRA* coronary magnetic resonance angiography, *CS* compressed sensing, *TR* repetition time, *TE* echo time, *FOV* field of view

### Data acquisition and image reconstruction of CS whole-heart CMRA with CS and DLR

First, PI consistently undersamples the k-space. In this study, we used autocalibrating reconstruction for Cartesian imaging (ARC) as PI [17]. A feature of the ARC is that the central part of the k-space is used for self-calibration, and unfolding is performed in the x-ky-kz domain. The ARC algorithm was supplied by the vendor.

Second, CS is a technique used for randomly undersampling the k-space [5]. The CS algorithm minimizes the L1 norm of the image after a sparse transformation by estimating the undersampled points. CS requires iterative calculations. A feature of CS is that it reduces the imaging time without reducing the SNR, which can be reduced by undersampling. The CS algorithm was supplied by the vendor. To estimate the undersampled k-space, we combine ARC with CS [18].

**Fig. 1:**
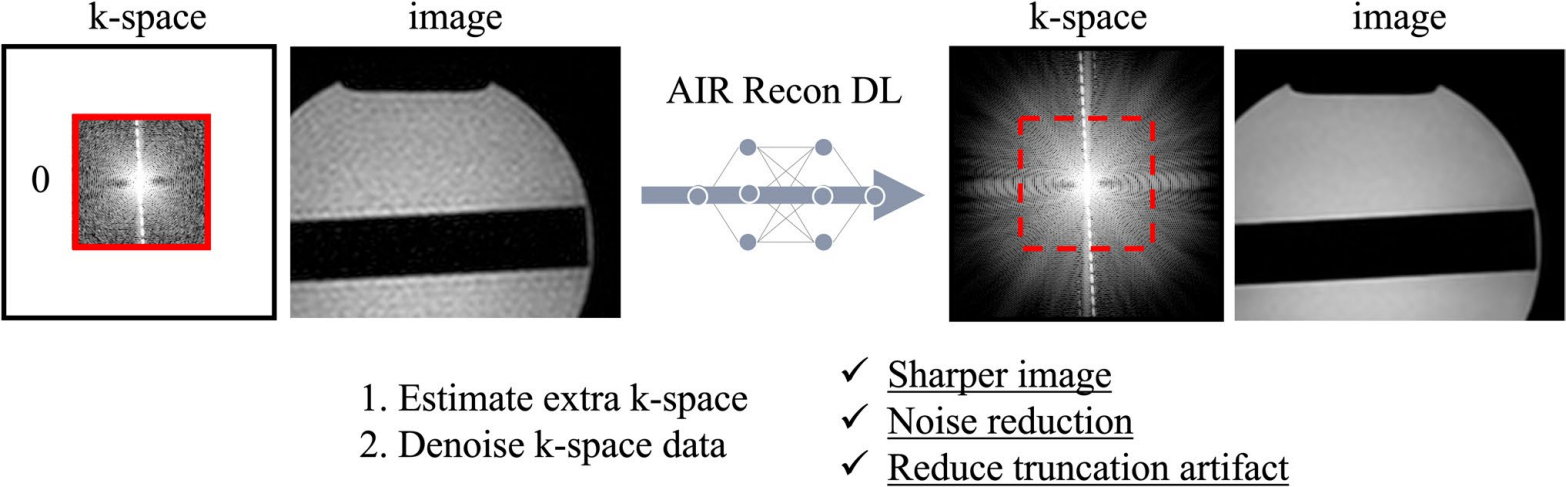
AIR Recon DL algorithm. AIR Recon DL estimates and complements high-spatial frequency information in k-space filled with zeros by zero-fill interpolation processing. In addition, AIR Recon DL denoises k-space data. Thus, AIR Recon DL sharpens images, reduces noise and truncation artifacts

Third, AIR Recon DL was used as the DLR method [19]. AIR Recon DL performs DLR in the k-space domain rather than in the image domain (Fig. 1). When converting k-space data into an image, a Lorentzian-shaped k-space filter is sometimes used to reduce aliasing artifacts; however, this reduces image sharpness. AIR Recon DL fulfills the outer regions of the k-space without using a k-space filter. This simultaneously reduces aliasing artifacts and improves sharpness and SNR. The research version of the AIR Recon DL algorithm was provided by a vendor. The product version of the AIR Recon DL can be used for PI but not for CS. The research version of the AIR Recon DL can be used for PI and CS. The product and research versions use nearly identical principles. Product version uses Graphic Processor, and post-processing can be done automatically. However, in the research version, post-processing must be done manually on a conventional processor on the host computer. In DLR, ARC, and CS, raw data are treated as complex-number data. AIR Recon DL was performed offline on CS CMRA data after scanning.

### Qualitative image assessment

**Fig. 2:**
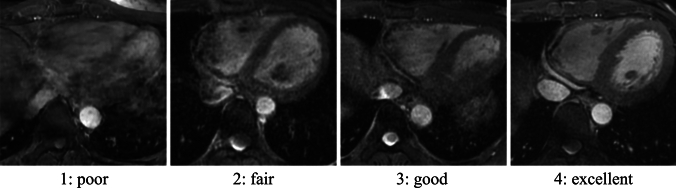
The 4-point subjective scale for qualitative image analysis

Two radiologists with 15 years (reader 1) and 5 years (reader 2) of experience in cardiac imaging independently assessed the qualitative image quality of the coronary arteries. Coronary arteries were assessed separately for the RCA, left anterior descending artery (LAD), and left circumflex artery (LCX). The assessment focused on the vessel sharpness and artifacts. Coronary arteries were assessed mainly in the proximal to middle locations. We used a 4-point subjective scale for qualitative image analysis: 4, excellent (vessel well visualized with sharply defined borders); 3, good (vessel adequately visualized with only mildly blurred borders); 2, fair (coronary vessel visible but with low confidence in diagnosis due to moderately blurred borders); and 1, poor (coronary vessel barely visible or obscured by noise) [6] (Fig. 2). All image quality assessments were performed in the axial orientation. The image quality assessed by reader 1 was used, and the inter-observer agreement with reader 2 was calculated for subsequent analysis.

### Quantitative image assessment

**Fig. 3:**
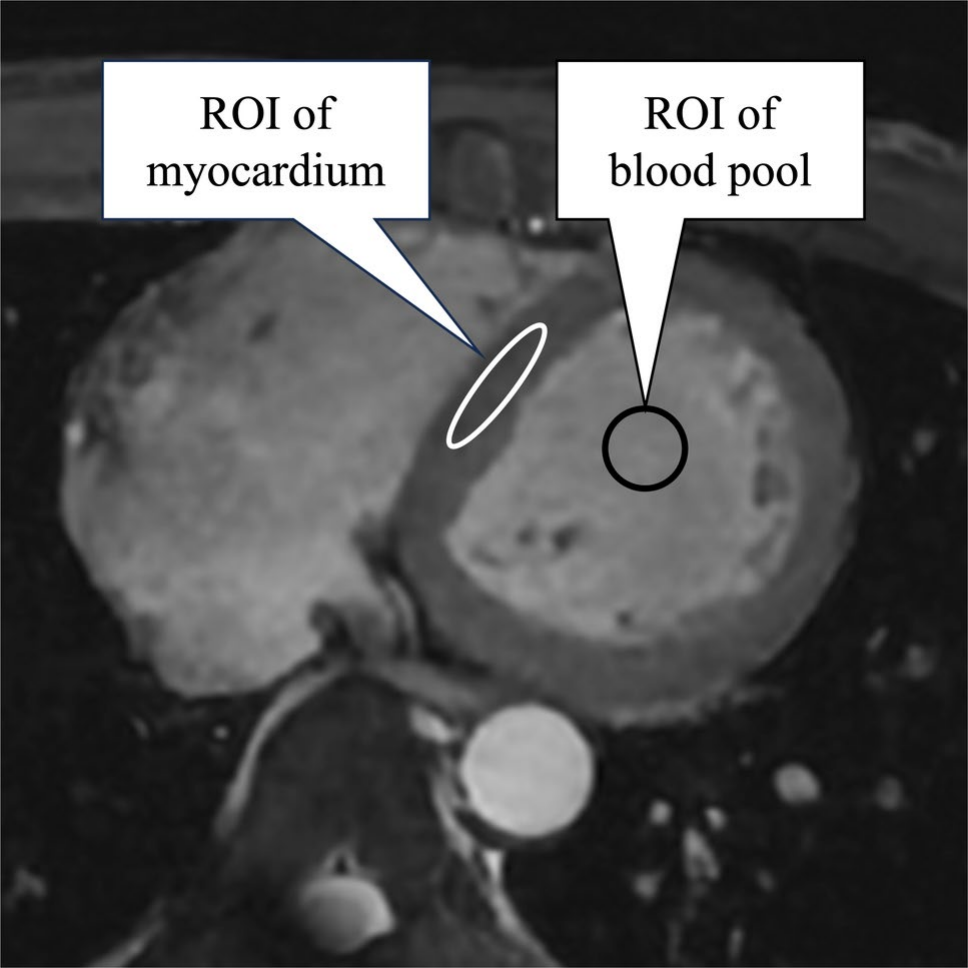
The ROIs placement for the calculation of signal-to-noise ratio and contrast-to-noise ratio. The ROIs were set without artifacts on the ventricular septum and left ventricular blood pool on the same slice. ROI, region of interest

Quantitative image assessment was performed by a radiologist with 5 years of experience. The CSAI, CS, and conventional CMRA images were assessed using the dedicated workstation (SYNAPSE VINCENT; Fujifilm Corp., Ltd., Tokyo, Japan). The regions of interest (ROIs; size: 80–100 mm^2^) were set without artifacts on the ventricular septum and left ventricular (LV) blood pool on the same slice to define the SNR and CNR (Fig. 3). The ROIs of the three images were set as much as possible at the same site. The SNR and CNR were defined using the following equation: SNR = signal intensity of the myocardium (SImyo)/standard deviation of the myocardium (SDmyo), CNR = (signal intensity of blood [SIblood]/standard deviation of the blood [SDblood])-(SImyo/SDmyo) [20].

Vessel sharpness in the RCA#1, LAD#6, and LCX#11 was evaluated using the following methods: The signal intensity profiles were obtained along a user-defined line perpendicular to the major axes of the vessel. The vessel sharpness was assessed by calculating the 20th and 80th percentile points between the maximum and background signal intensities for each side of the signal intensity profile. The distance between these two points was then determined for each side in millimeters. Vessel sharpness was defined as the reciprocal of the average distance between the two points [21].

### Statistical analysis

Statistical analyses were performed using a statistical software (JMP version 13; SAS Institute, Cary, North Carolina, USA). Continuous variables are presented as mean ± SD or median (first and third quartiles). Paired t tests were used to compare the acceptance rates. The Wilcoxon matched-pairs signed-rank test and Friedman test were used to compare the imaging times, image quality, SNR, CNR, and vessel sharpness. The quadratic-weighted kappa test was used to evaluate the inter-observer agreement of image quality (> 0.81, excellent agreement; 0.61–0.80, good agreement; 0.41–0.60, moderate agreement; 0.21–0.40, fair agreement; and < 0.20, poor agreement). A *P* value < 0.05, and Bonferroni correction was used to reduce the chance of false-positive results (type I error) when multiple pairwise tests were performed.

## Results

All CS and conventional CMRA scans were successfully performed in all the volunteers. The coronary arteries of all volunteers were analyzed. All volunteers had a regular sinus rhythm with a mean heart rate of 65.2 ± 13.7 bpm (range, 45–92 bpm). The respiratory acceptance rate was 50.6 ± 12.5% (range, 32.6–73.4%) for CS CMRA, and 52.2 ± 14.0% (range, 27.4–78.8%) for conventional CMRA (*p* = 0.89). The median total imaging time was 460 s (343, 753 s) for CS and 727 s (567, 939 s) for conventional CMRA (*p* < 0.001).

### Qualitative image assessment

**Fig. 4:**
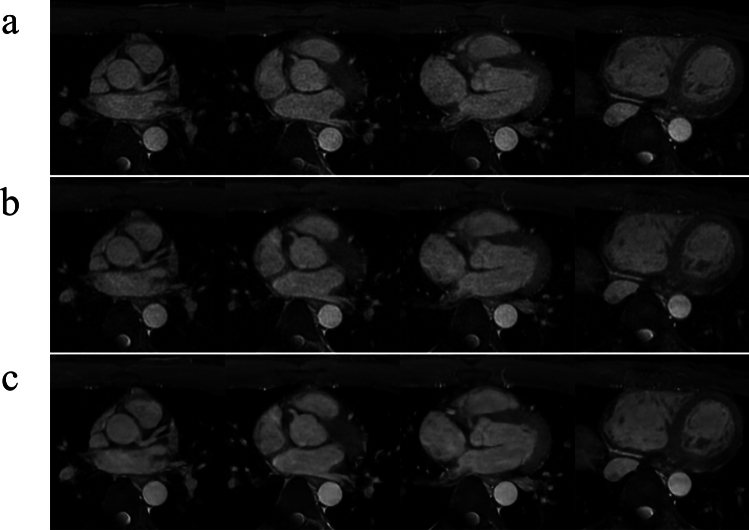
Representative axial images of conventional (**a**), CS (**b**), and CSAI (**c**) CMRA acquired from a 35-year-old male. The image quality of each coronary arteries was rated as excellent (i.e., score of 4) for conventional and CSAI CMRA and as good (i.e., score of 3) for CS CMRA. CMRA, coronary magnetic resonance angiography; CS, compressed sensing; CSAI, compressed sensing and artificial intelligence

**Fig. 5:**
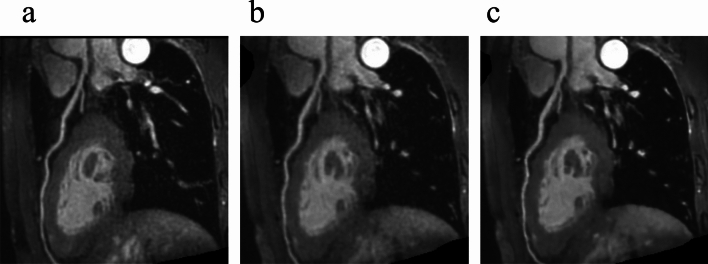
Representative curved planar reconstruction for left anterior descending artery of conventional (**a**), CS (**b**), and CSAI (**c**) CMRA acquired from a 34-year-old male. The image quality, as assessed by axial images, was excellent (i.e., score of 4) for conventional and CSAI CMRA and good (i.e., score of 3) for CS CMRA. *CMRA* coronary magnetic resonance angiography, *CS* compressed sensing, *CSAI* compressed sensing and artificial intelligence

Table 2 summarizes the image quality scores for each coronary artery. The LAD and LCX scores were significantly higher in conventional CMRA and CSAI CMRA than in CS CMRA (LAD: conventional CMRA vs. CS CMRA, *p* = 0.023; CSAI CMRA vs. CS CMRA, *p* < 0.001; conventional CMRA vs. CSAI CMRA, *p* = 0.117; LCX: conventional CMRA vs. CS CMRA, *p* = 0.047; CSAI CMRA vs. CS CMRA, *p* = 0.002; conventional CMRA vs. CSAI CMRA, *p* = 0.680); however, the RCA scores did not vary among the three groups (*p* = 0.087). The two radiologists showed good agreement in subjective image quality scores (Kappa = 0.78 for CSAI CMRA, Kappa = 0.72 for CS CMRA, Kappa = 0.72 for conventional CMRA). Representative examples are shown in Fig. 4 and 5.

**Table 2:** Qualitative assessment

	Conventional CMRA	CS CMRA	CSAI CMRA	P value
RCA	3.0 ± 0.8	2.6 ± 0.6	3.0 ± 0.8	0.087
LAD	3.3 ± 0.7	2.9 ± 0.6	3.7 ± 0.6	< 0.001
LCX	3.3 ± 0.7	2.9 ± 0.6	3.5 ± 0.7	< 0.001

The data are presented as the mean ± standard deviation

P values are the results of the Friedman test

*CMRA* coronary magnetic resonance angiography, *CS* compressed sensing, *CSAI* compressed sensing and artificial intelligence, *RCA* right coronary artery, *LAD* left anterior descending artery, *LCX* left circumflex artery

### Quantitative vessel analysis

**Fig. 6:**
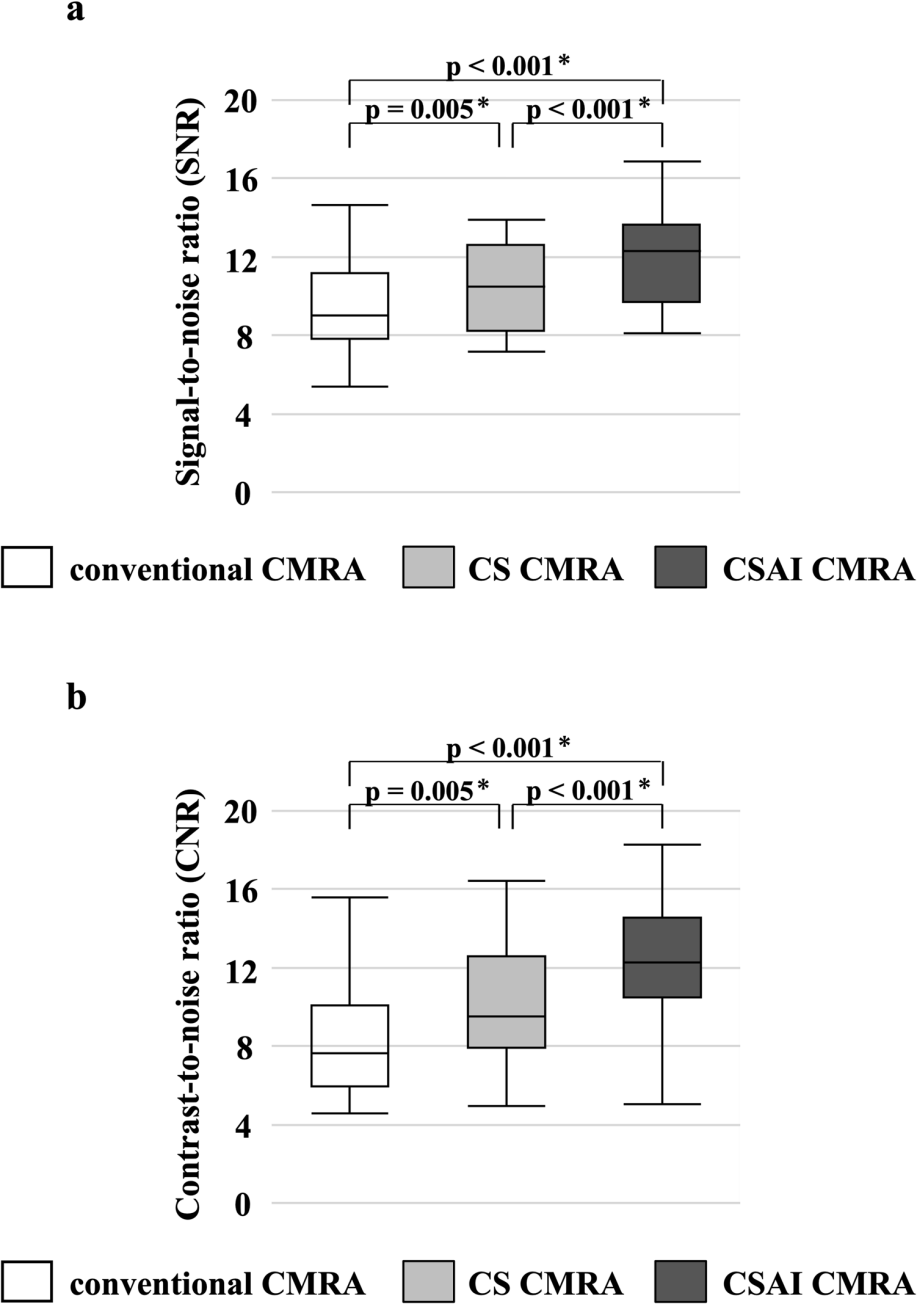
**a** The SNR for the three techniques. **b** The CNR for the three techniques. *SNR* signal-to-noise ratio, *CNR* contrast-to-noise ratio, *CMRA* coronary magnetic resonance angiography, *CS* compressed sensing, *CSAI* compressed sensing and artificial intelligence

**Fig. 7:**
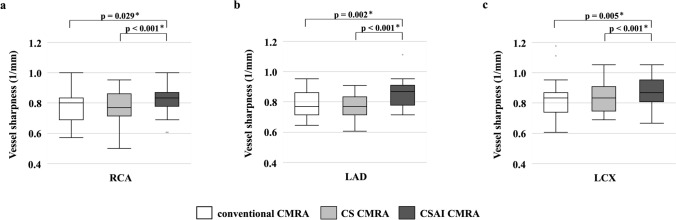
The vessel sharpness for the three techniques (**a**: right coronary artery, **b**: left anterior descending artery, **c**: left circumflex artery). *CMRA* coronary magnetic resonance angiography, *CS* compressed sensing, *CSAI* compressed sensing and artificial intelligence

The SNR and CNR were significantly higher in CSAI CMRA, and those in CS CMRA were higher than those in conventional CMRA (Table 3, Fig. 6). Vessel sharpness was significantly higher in CSAI CMRA, with no significant difference between CS CMRA and conventional CMRA (Table 3, Fig. 7).

**Table 3:** Quantitative assessment

	Conventional CMRA	CS CMRA	CSAI CMRA
SNR	9.0 (7.8, 11.1)	10.5 (8.2, 12.6)	12.3 (9.7, 13.7)
CNR	7.7 (6.0, 10.1)	9.5 (7.9, 12.6)	12.3 (10.5, 14.5)
Vessel sharpness (1/mm)			
RCA	0.80 (0.69, 0.83)	0.77 (0.71, 0.86)	0.83 (0.78, 0.90)
LAD	0.77 (0.71, 0.86)	0.77 (0.71, 0.83)	0.87 (0.78, 0.91)
LCX	0.83 (0.74, 0.87)	0.83 (0.75, 0.91)	0.87 (0.80, 0.95)

The data are presented as the median (first quartile, third quartile)

*CMRA* coronary magnetic resonance angiography, *CS* compressed sensing, *CSAI* compressed sensing and artificial intelligence, *SNR* signal-to-noise ratio, *CNR* contrast-to-noise ratio, *RCA* right coronary artery, *LAD* left anterior descending artery, *LCX* left circumflex artery

## Discussion

In this prospective study, we validated the usefulness of non-contrast CSAI CMRA. These results suggest that non-contrast CSAI MRA has the potential to shorten the imaging time for non-contrast CMRA while maintaining good image quality.

CMRA is a noninvasive method that can evaluate the coronary artery morphology and function without radiation exposure or contrast administration [3]. CMRA has the disadvantage of long imaging times; therefore, research is underway to shorten the imaging time by undersampling at the time of the scan. One such high-speed imaging method is PI, which has become an indispensable technique in clinical practice. PI can shorten imaging times by collecting data in k-space with regular data thinning using the sensitivity encoding of multiple receiver coils for recovering acquired data [22–24]. The disadvantages of PI include the limited maximum acceleration factor owing to the number of receiving channels and peculiar artifacts such as residual aliasing and increased g-factor noise [14]. Another high-speed imaging method, CS, has gained popularity in recent years. CS performs random sampling from the original data and converts it into sparse data. Subsequently, a denoising calculation is repeatedly performed in the sparse data, and a less noisy image close to the full data is reconstructed [5, 25]. CS and PI CMRA at the same acceleration factor in previous reports were significantly higher upon qualitative evaluation of image quality and SNR for CS CMRA [26].

AI-based technologies have recently been developed for cardiac MRI. Various DLR techniques have reportedly improved the image quality and shortened imaging times for cine and CMRA [27–30]. The AIR Recon DL used in this study is an image reconstruction pipeline that includes a deep convolutional neural network to overcome the limitations of conventional reconstruction pipelines, namely ineffective denoising, image blurring, and residual ringing.

In our study, CS CMRA significantly shortened the imaging time when compared with conventional CMRA. The image quality scores were significantly lower for CS CMRA than for conventional CMRA. These results are similar to those reported by Nakamura et al. [6]. CS shortened the imaging time by randomly thinning and collecting data; however, we believe that this reduced the number of data samples and degraded the image quality. Although CS-CMRA had reduced noise, it was slightly more pixelated and textured in appearance than conventional CMRA because of noise reduction in the reconstruction step. This may have affected the image quality evaluation. Adding DLR to CS CMRA improved the image quality compared with that of conventional CMRA. CSAI CMRA had an image quality score comparable to that of conventional CMRA, indicating that adding DLR to CS CMRA improved the image quality.

The CS CMRA had a higher SNR and CNR than conventional CMRA. These results are attributed to the CS noise reduction process. AIR Recon DL was able to further reduce noise in the k-space data, further improving the SNR and CNR of CSAI CMRA compared with those of CS CMRA. AIR Recon DL is unique in that it performs DLR in the k-space domain rather than in the image domain. AIR Recon DL trained on high resolution MRI k-space dates with various MR contrasts and high SNR. By training on these data, AIR Recon DL optimized a machine learning model to improve SNR, reduce truncation artifacts, and sharpen low-resolution, low SNR images [19].

Edge sharpness was not significantly different between CS CMRA and conventional CMRA. The low spatial frequency information at the center of the k-space determines the signal intensity or image contrast, whereas the outer high spatial frequency information determines the image resolution [31]. Low-spatial-frequency information in k-space is dense, whereas high-spatial-frequency information is sparse. In conventional CMRA, data for high spatial frequency information are not collected and filled with zeros to shorten the imaging time [32–34]. CS only reduces noise in the low spatial frequency information of the k-space and does not affect the high spatial frequency information of the k-space. AIR Recon DL estimates the high spatial frequency information of a k-space filled with zeros and complements it to achieve image sharpening [19]. Thus, the sharpness of the coronary arteries improved only with CSAI-MRA.

Our study included healthy volunteers, and the mean acceptance rate was relatively high. However, patients with cardiac disease presumably have lower acceptance rates and longer examination times owing to the unstable respiratory status associated with the underlying disease and fatigue from prolonged scanning. We believe that the benefit of shortening the CS time increases as the imaging time increases.

Our study used non-contrast CMRA because it was performed in healthy volunteers. The optimal non-contrast CMRA imaging technique differs between 1.5 T MRI and 3 T MRI. The bSSFP sequence is common in 1.5 T CMRA [3]. In bSSFP, multiple transverse magnetization components are combined in a steady-state to produce a strong signal [35]. The contrast of bSSFP depends on the T2/T1 ratio. The relatively high T2/T1 ratio of blood also makes bSSFP suitable for MRA. However, the use of bSSFP at 3 T MRI is limited owing to several reasons: increased magnetic field (B₀) and radiofrequency (B₁) inhomogeneities, degraded image quality caused by RF pulse-induced dielectric effects, and increased power deposition in the human body at 3 T limits the use of large flip angles [36]. Therefore, 3 T CMRA uses a spoiled GRE sequence that is resistant to magnetic field inhomogeneity, but the signal in the coronary arteries is lower than SSFP in non-contrast. 3 T CMRA using spoiled GRE sequences significantly improves blood contrast during contrast agent administration, so contrast agent is commonly used in 3 T CMRA. In the CS technique, the contrast in the image plays a major role in its ability to reconstruct vastly undersampled images. A high contrast often results in large distinct sparse coefficients [5]. Therefore, contrast-enhanced CS CMRA is expected to provide better image quality than non-contrast CS CMRA. Although the CS acceleration factor in our study was relatively low, image quality can presumably be maintained even with an increased CS acceleration factor, in contrast to CMRA, which may further shorten the imaging time.

Our study had certain limitations. First, the study included a small number of volunteers. CSAI MRA is a new trial, and as a preliminary study, it was first conducted on 20 volunteers to evaluate whether it could shorten imaging time and improve image quality. We plan to increase the number of cases and study them in clinical cases to validate our study findings. Second, Vascular stenosis was not evaluated in this preliminary study. Ogawa et al. reported the effectiveness of CS CMRA in detecting coronary artery stenosis; however, the effectiveness of CS combined with DLR in detecting coronary artery stenosis using CMRA has not yet been fully investigated [37]. Several DLRs have been studied in addition to the AIR Recon DL used in this study, all of which have great potential for use in the scan and reconstruction of CMRA images; however, no consensus exists on which technique is most advantageous [28–30]. Further studies are warranted on DLR, including its ability to detect coronary artery stenosis. Third, it is possible that g-factor affected the SNR and CNR results. PI uses multiple array coils for imaging and uses the difference in coil sensitivity distribution to expand aliasing artifacts. Low intercoil sensitivity independence in this expansion process amplifies the noise, the degree of which depends on the location on the image and is called the g-factor [38]. The g-factor causes position dependence in SNR and CNR. In measuring SNR and CNR, the ROIs placement for CSAI, CS, and conventional CMRA were identical to the extent possible; however, the g-factor may have affected the results. Fourth, AIR Recon DL was performed offline. The use of AIR Recon DL online with the ability to automatically be reconstructed after CMRA imaging will further promote its use in clinical practice. It is hoped that overcoming of these challenges will reduce the burden on patients undergoing CMRA.

## Conclusion

Non-contrast CMRA using CS combined with DLR could shorten the imaging time when compared with conventional CMRA, while maintaining good image quality. Further studies are warranted to evaluate the utility of this technique in clinical settings.

## Conflict of interest

Mitsuharu Miyoshi is an employee of GE HealthCare. The other authors have declared no conflicts of interest.

## Ethical approval

All procedures performed in studies involving human participants were conducted in accordance with the ethical standards of the institutional and/or national research committee and with the 1964 Helsinki declaration and its later amendments or comparable ethical standards. This prospective study was approved by our institutional review board (Institutional Review Board, Ehime University Hospital No.2311006).

## Informed consent

Informed consent was obtained from all individual participants included in the study.
